# A single-oral bolus of 100,000 IU of cholecalciferol at hospital admission did not improve outcomes in the COVID-19 disease: the COVID-VIT-D—a randomised multicentre international clinical trial

**DOI:** 10.1186/s12916-022-02290-8

**Published:** 2022-02-18

**Authors:** Jorge B. Cannata-Andía, Augusto Díaz-Sottolano, Pehuén Fernández, Carmen Palomo-Antequera, Pablo Herrero-Puente, Ricardo Mouzo, Natalia Carrillo-López, Sara Panizo, Guillermo H. Ibañez, Carlos A. Cusumano, Carolina Ballarino, Vicente Sánchez-Polo, Jacqueline Pefaur-Penna, Irene Maderuelo-Riesco, Jesús Calviño-Varela, Mónica D. Gómez, Carlos Gómez-Alonso, John Cunningham, Manuel Naves-Díaz, Walter Douthat, José L. Fernández-Martín, the COVID-VIT-D trial collaborators

**Affiliations:** 1 Hospital Universitario Central de Asturias (HUCA), Avda. Roma s/n., 33011 Oviedo, Spain; 2 Instituto de Investigación Sanitaria del Principado de Asturias (ISPA), Oviedo, Spain; 3 Universidad de Oviedo, Oviedo, Spain; 4 Retic REDinREN-ISCIII, Madrid, Spain; 5 Centro de Salud Roces Montevil, Gijón, Spain; 6 Hospital Privado Universitario de Córdoba, Córdoba, Argentina; 7 Hospital Raúl Ángel Ferreyra, Córdoba, Argentina; 8 Instituto Universitario de Ciencias Biomédicas de Córdoba (IUCBC), Córdoba, Argentina; 9 Hospital Universitario El Bierzo, Ponferrada, Spain; 10 Hospital Independencia, Santiago del Estero, Argentina; 11 Instituto de Nefrología Pergamino SRL, Pergamino, Argentina; 12 Hospital Militar Central Cirujano Mayor Dr. Cosme Argerich, Buenos Aires, Argentina; 13 Hospital General de Enfermedades del Instituto Guatemalteco de Seguridad Social (IGSS), Ciudad de Guatemala, Guatemala; 14 Hospital Barros Luco Trudeau, Santiago, Chile; 15 Universidad de Chile, Santiago, Chile; 16 Hospital Universitario San Agustín (HUSA), Avilés, Spain; 17 Hospital Lucus Augusti, Lugo, Spain; 18 Hospital Julio C. Perrando, Resistencia, Argentina; 19 Centre for Nephrology, Royal Free Hospital and University College London, London, UK

**Keywords:** SARS-CoV-2, COVID-19 disease, Cholecalciferol, Vitamin D

## Abstract

**Background:**

Vitamin D status has been implicated in COVID-19 disease. The objective of the COVID-VIT-D trial was to investigate if an oral bolus of cholecalciferol (100,000 IU) administered at hospital admission influences the outcomes of moderate-severe COVID-19 disease. In the same cohort, the association between baseline serum calcidiol levels with the same outcomes was also analysed.

**Methods:**

The COVID-VIT-D is a multicentre, international, randomised, open label, clinical trial conducted throughout 1 year. Patients older than 18 years with moderate-severe COVID-19 disease requiring hospitalisation were included. At admission, patients were randomised 1:1 to receive a single oral bolus of cholecalciferol (*n*=274) or nothing (*n*=269). Patients were followed from admission to discharge or death. Length of hospitalisation, admission to intensive care unit (ICU) and mortality were assessed.

**Results:**

In the randomised trial, comorbidities, biomarkers, symptoms and drugs used did not differ between groups. Median serum calcidiol in the cholecalciferol and control groups were 17.0 vs*.* 16.1 ng/mL at admission and 29.0 vs*.* 16.4 ng/mL at discharge, respectively. The median length of hospitalisation (10.0 [95%CI 9.0–10.5] vs*.* 9.5 [95%CI 9.0–10.5] days), admission to ICU (17.2% [95%CI 13.0–22.3] vs. 16.4% [95%CI 12.3–21.4]) and death rate (8.0% [95%CI 5.2–12.1] vs*.* 5.6% [95%CI 3.3–9.2]) did not differ between the cholecalciferol and control group. In the cohort analyses, the highest serum calcidiol category at admission (>25ng/mL) was associated with lower percentage of pulmonary involvement and better outcomes.

**Conclusions:**

The randomised clinical trial showed the administration of an oral bolus of 100,000 IU of cholecalciferol at hospital admission did not improve the outcomes of the COVID-19 disease. A cohort analysis showed that serum calcidiol at hospital admission was associated with outcomes.

**Trial registration:**

COVID-VIT-D trial was authorised by the Spanish Agency for Medicines and Health products (AEMPS) and registered in European Union Drug Regulating Authorities Clinical Trials (EudraCT 2020-002274-28) and in ClinicalTrials.gov (NCT04552951).

**Supplementary Information:**

The online version contains supplementary material available at 10.1186/s12916-022-02290-8.

## Background

The “classical effects” of vitamin D on the bone and mineral metabolism are well established [1, 2]. However, in the last two decades, many “non-classical” actions of vitamin D on the immune system [3] that may contribute to a better defensive response against several bacterial and viral infections have been described [4–7].

Deficiency of vitamin D, assessed by serum calcidiol levels, is common, particularly in the elderly and frail, and it has been associated with higher morbidity and mortality [8–12]. The information on a possible beneficial role of vitamin D comes from randomised trials, experimental and clinic-epidemiological association studies, and reviews [13–20]. The meta-analyses of randomised clinical trials on vitamin D and respiratory infections and chronic diseases show no consensus on the effects of vitamin D supplementation [21–23].

Therefore, the COVID-VIT-D trial was designed to investigate if a single oral bolus of 100,000 IU of cholecalciferol administered at hospital admission could influence the outcomes of patients with COVID-19 disease. In addition, the study also aimed to find out if vitamin D status at hospital admission (serum calcidiol concentration) influenced the pulmonary involvement at admission and the outcomes of the disease.

## Methods

### Study design and dosing

The COVID-VIT-D was a randomised, open label, multicentre, international clinical independent trial designed and coordinated by the Bone and Mineral Research Unit of Hospital Universitario Central de Asturias (HUCA), Oviedo, Spain, carried out in 12 centres from four countries (Spain, Argentina, Guatemala and Chile), not supported by any pharmaceutical company. In clinical practice, the current dose of cholecalciferol used in different countries to maintain optimal serum calcidiol levels with no risk of hypercalcemia, either as a dietary supplement or as a prescribed supplement, ranged between 15,000 and 50,000 IU, administered daily or monthly. Thus, in order to achieve the optimal serum calcidiol levels in a few days [23], minimising the risks of hypercalcaemia [24–28], in agreement with the Spanish Agency for Medicines and Health products (AEMPS), which is part of the European Agency of Medicines (AEM), responsible for the authorization of clinical trials**,** it was decided to administer a single oral bolus of 100,000 IU of cholecalciferol.

### Participants

**Fig. 1:**
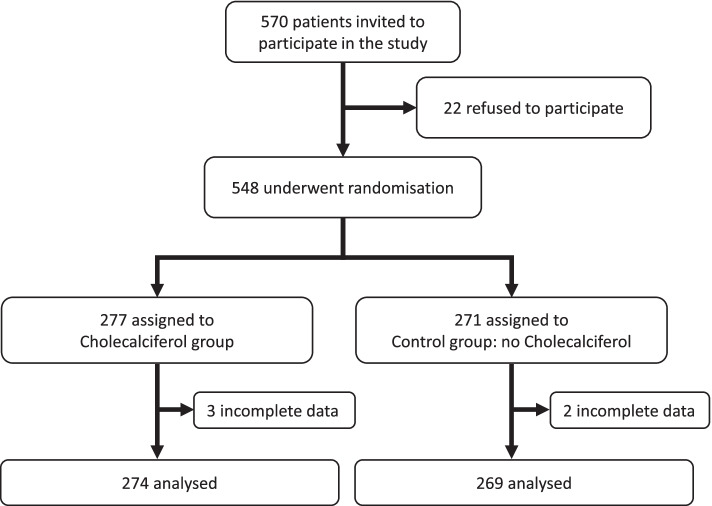
COVID-VIT-D trial flow chart

Eligible participants were aged 18 years or above requiring hospitalisation for moderate-severe COVID-19 disease who consented the participation in the study, 570 patients were invited to participate (Fig. 1), finally 543 patients (cholecalciferol *n*=274, control *n*=269) from four countries that were admitted and discharged from hospital since April 4, 2020, to April 22, 2021, were analysed (Argentina; six centres *N*=295, Spain; four centres *N*=173, Guatemala; one centre *N*=47, Chile; one centre *N*=28). Patients with dementia or not able to communicate, tested negative for Severe Acute Respiratory Syndrome Coronavirus 2 (SARS-CoV-2) despite clinical findings compatible with COVID-19 disease, pregnant and lactating women, patients who received any form of vitamin D in the previous 3 months and allergic to vitamin D were excluded.

### Criteria for hospitalisation/intensive unit care admission

Criteria for hospitalisation were radiological evidence of pulmonary involvement compatible with the COVID-19 disease (bilateral multifocal ground-glass opacities > 50%), and/or moderate-severe flu-like syndrome having oxygen saturation lower than 94% breathing room air and/or additional risk factors (hypertension, diabetes, chronic pulmonary and cardiac diseases, or other serious risk factors). Criteria for intensive care unit (ICU) admission were oxygen saturation lower than 93% on high flow oxygen therapy with FiO_2_ of 70% and/or severe haemodynamic instability.

### Ethics considerations

The ethics committees of all participating centre approved the study. Due to the COVID-19 pandemic and in order to avoid unnecessary exposure to the SARS-CoV-2 virus, all ethics committees authorised verbal consent. The trial was conducted according to the ethical principles of the Declaration of Helsinki.

### Data collection and randomisation

At the time of hospital admission, serum calcidiol and other biomarkers were measured (Table 1). Patients were randomised to receive a single oral bolus of 100,000 IU of cholecalciferol, (cholecalciferol group) or nothing (control group). The case sheet of each patient included in the study had a note informing the patient was included in the COVID-VIT-D trial, but there was no information about the arm in which the patient was included (active or control). This information was withheld in the list of randomisation of each centre. Furthermore, the serum calcidiol levels at admission was blinded for the medical staff who managed the patients. All patients received other therapies according to local protocols. Randomisation was performed individually in each centre using a computer-generated list with a 1:1 ratio, and data included in the study were collected in a database. The text of the verbal consent, the database in which patients were identified using different numbers per each centre and patient, and the randomisation lists of the 12 centres were produced and distributed by the HUCA coordinating centre which monthly received the updated database from all participating centres.

**Table 1:** Variables collected in the COVID-VIT-D trial included in this report

Demographic and comorbidities	
Date of birth	Diabetes (yes/no)
Gender (male/female)	Cardiovascular disease (yes/no)
Height (cm)	Hypertension (yes/no)
Weight (kg)	Asthma (yes/no)
	COPD (yes/no)
Hospitalisation (clinical and evolutive data)	
Hospital admission date	Death date
Hospital discharge date	
Biochemical and imaging parameters at admission and discharge	
Calcidiol (ng/mL)	Interleukin-6 (pg/mL)
CRP (mg/dL)	Ferritin (ng/mL)
Albumin (g/L)	Calcium (mg/dL)
Haemoglobin (g/dL)	Phosphate (mgl/dL)
LDH (U/L)	X-Ray/CAT (positive/ doubtful/negative)
Leucocytes (No./μL)	
Types of drugs received during the hospitalisation	
Cholecalciferol (yes/no)	Enoxaparin (yes/no)
Azithromycin (yes/no)	Methylprednisolone (yes/no)
Ceftriaxone (yes/no)	Dexamethasone (yes/no)

*COPD* Chronic obstructive pulmonary, *CRP* C-reactive protein, *CAT* Computed axial tomography

### Follow-up

Patients were followed from hospital admission to discharge or death during their hospitalisation period; there was no follow-up after the hospital discharge. Demographics, comorbidities, symptoms, biochemical parameters, chest X-ray and/or computed axial tomography, clinical evolutionary data, types of therapy received during the hospitalisation, admission to ICU and death were collected in the database. The data used in this report were those necessary for the present analyses (29 variables and 14 items, Table 1), selected from the complete database distributed to all centres, which included 53 variables and 38 items (Additional file 1: Table S1).

### Outcomes

The end points of the COVID-VIT-D trial were three outcomes of the COVID-19 disease: length of hospitalisation, admission to the ICU and mortality. In the cohort analyses, the relationship between serum calcidiol at admission with (a) pulmonary involvement and (b) with the same three outcomes of the trial was assessed.

### Clinical trial registration

The COVIT-VIT-D was authorised as a low-intervention clinical trial by the AEMPS and registered in the European Union Drug Regulating Authorities Clinical Trials (EudraCT 2020-002274-28) and in ClinicalTrials.gov (NCT04552951). Protocol details can be found in the Additional file 2 [1, 3, 5, 6, 8, 10–13, 17, 18, 29–53].

### Laboratory analyses and imaging techniques

Serum calcidiol was measured locally in each centre by electrochemiluminiscence (Cobas e601/e801, Roche Diagnostics) or chemiluminiscence immunoassay (Architect 2000, Abbott and Atellica Solution, Siemens). C-reactive protein (CRP), albumin, lactate dehydrogenase, interleukin-6 (IL-6), haemoglobin, leucocytes, ferritin, calcium and phosphate were measured by autoanalyser (Roche diagnostics, Mindray, Beckman Coulter, Wiener lab, BioMérieux, Abbott, Werfen, Radiometer and Siemens). SARS-CoV-2 status was investigated in nasopharyngeal swabs using either polymerase chain reaction test (PCR) or antigen tests.

Pulmonary involvement was evaluated by pulmonary X-ray and/or pulmonary computed axial tomography (CAT). In the database three categories were considered: positive (pneumonia), negative (no pneumonia) and doubtful (not clearly positive but not normal) (Table 1). In this analysis, doubtful patients were considered positive.

### Statistical analyses

Continuous variables were described by using median and interquartile range (IQR), and categorical variables were summarised using absolute and relative frequencies. Differences between groups were tested using the Kruskal-Wallis or Mann-Whitney test for continuous variables, and chi-squared test or Fisher’s exact test (frequencies less than five), for categorical variables.

Patients were described according to initial calcidiol levels (≤10, 10–15, 15–20, 20–25 and >25 ng/mL). The association between the serum calcidiol levels at hospital admission and length of hospitalisation was assessed using linear regression analysis. Binary logistic regression was used to study the association between calcidiol levels and pulmonary involvement and Cox regression was used for admission to ICU, and mortality. Multivariate adjustments with ten variables: demographics (*N*=2), comorbidities (*N*=5) and serum biochemical parameters (*N*=3) were performed in patients in whom at least 70% of these variables were collected. A complete set of gender, age-matched and control group analyses were performed. All statistical analyses were done using R statistical software version 4.0.4.

#### Role of the funding source

This study was not supported by any pharmaceutical company.

## Results

### Comparison between the cholecalciferol and control group

The demographics and comorbidities are shown in Table 2. Overall, the median age was 58.0 years (Argentina 57.0, Spain 62.0, Guatemala56.0, Chile61.5), and the 65.0% were males. Hypertension (43.8%), diabetes (24.7%) and cardiovascular disease (21.2%) were the most frequent comorbidities. Pulmonary involvement was diagnosed in 83.1% of the admitted patients. Fever (71.5%), cough (66.5%), weakness (62.2%), dyspnoea (54.0%) and headache (34.6%) were the most frequent symptoms.

**Table 2:** Demographic parameters, comorbidities, pulmonary involvement and symptoms at admission

	Cholecalciferol group		Control group	
	***n***	***n***=274	***n***	***n***=269
**Demographics**				
Age (years), median [IQR]	274	59.0 [49.0, 70.0]	269	57.0 [45.0, 67.0]
Males, *n* (%)	274	181 (66.1)	269	172 (63.9)
BMI (Kg/m^2^), median [IQR]	214	28.3 [25.7, 30.9]	207	28.7 [25.9, 32.4]
Smokers, *n* (%)	272	31 (11.4)	269	29 (10.8)
**Comorbidities**				
Hypertension, *n* (%)	274	114 (41.6)	269	124 (46.1)
Diabetes, *n* (%)	274	58 (21.2)	269	76 (28.3)
Cardiovascular disease, *n* (%)	274	55 (20.1)	269	60 (22.3)
Asthma, *n* (%)	274	14 (5.1)	269	16 (5.9)
COPD, *n* (%)	274	14 (5.1)	269	9 (3.3)
**Pulmonary involvement,** ***n*** **(%)**^**a**^	274	234 (85.4)	269	217 (80.7)
**Symptoms**				
Fever, *n* (%)	274	190 (69.3)	269	198 (73.6)
Cough, *n* (%)	274	185 (67.5)	269	176 (65.4)
Weakness, *n* (%)	274	167 (60.9)	269	171 (63.6)
Dyspnoea, *n* (%)	274	150 (54.7)	269	143 (53.2)
Headache, *n* (%)	274	93 (33.9)	269	95 (35.3)
Anosmia, *n* (%)	274	46 (16.8)	269	61 (22.7)
Diarrhoea, *n* (%)	274	45 (16.4)	269	60 (22.3)
Ageusia, *n* (%)	274	37 (13.5)	269	40 (14.9)
Other, *n* (%)	274	52 (19.0)	269	57 (21.2)
Number of symptoms, median [IQR]	274	3.0 [2.0, 5.0]	269	4.0 [2.0, 5.0]

*n* number of patients available for analysis, *IQR* interquartile range, *COPD* chronic obstructive pulmonary disease

^a^Assessed by chest X-ray and/or computed axial tomography

The biochemical parameters at admission are depicted in Table 3. Median serum calcidiol did not differ by sex, but differences by countries were observed (Argentina16.0, Spain 13.4, Guatemala24.1, Chile 19.5 ng/mL). Table 4 shows the percentages of different types of drugs received during hospitalisation.

**Table 3:** Biochemical parameters at admission

	Cholecalciferol group		Control group	
	***n***	***n***=274	***n***	***n***=269
**Laboratory parameters**				
Calcidiol (ng/mL), median [IQR]	273	17.0 [11.8, 22.0]	265	16.1 [11.5, 22.0]
Creatinine (mg/dL), median [IQR]	269	0.9 [0.8, 1.1]	257	0.9 [0.8, 1.1]
CRP (mg/dL), median [IQR]	241	10.1 [4.4, 37.7]	239	8.9 [3.3, 25.9]
Albumin (g/L), median [IQR]	154	38.0 [34.3, 40.0]	146	39.0 [36.0, 41.0]
Haemoglobin (g/dL), median [IQR]	270	13.8 [12.9, 14.5]	262	14.0 [13.0, 14.9]
LDH (U/L), median [IQR]	229	382.0 [265.0, 491.0]	215	345.0 [244.5, 457.5]
Leucocytes (No./μL), median [IQR]	270	7.0 [5.4, 9.4]	262	7.0 [5.1, 8.8]
Interleukin-6 (pg/mL), median [IQR]	97	13.0 [6.0, 30.0]	93	11.0 [3.7, 25.7]
Ferritin (ng/mL), median [IQR]	229	750.0 [390.0, 1500.0]	220	587.5 [305.8, 1126.0]
Calcium (mg/dL), median [IQR]	204	8.8 [8.4, 9.2]	192	8.9 [8.5, 9.2]
Phosphate (mg/dL), median [IQR]	161	3.3 [2.8, 3.9]	151	3.2 [2.7, 3.8]

*n* number of patients available for analysis, *IQR* interquartile range, *CRP* C-reactive protein, *LDH* Lactate dehydrogenase

**Table 4:** Types and number of drugs received during the hospitalisation

	Cholecalciferol group		Control group	
	***n***	***n***=274	***n***	***n***=269
**Drugs prescribed**				
Cholecalciferol, *n* (%)	274	274 (100.0)	269	0 (0.0)
Enoxaparin, *n* (%)	270	210 (77.8)	264	191 (72.3)
Ceftriaxone, *n* (%)	271	100 (36.9)	264	94 (35.6)
Methylprednisolone, *n* (%)	271	99 (36.5)	265	94 (35.5)
Azithromycin, *n* (%)	272	88 (32.4)	265	97 (36.6)
Dexamethasone, *n* (%)	272	83 (30.5)	265	78 (29.4)
Number of drugs per patient, median [IQR]	272	2.0 [2.0, 3.0]	265	2.0 [2.0, 3.0]

The therapies used in less than 10% of patients (hydroxychloroquine *N*=48, lopinavir, ritonavir *N*=36, tocilizumab *N*=25 or plasma from convalescent patients *N*=53) were not included in the table

*n* number of patients available for analysis, *IQR* interquartile range

#### Effect of cholecalciferol on the outcomes

**Fig. 2:**
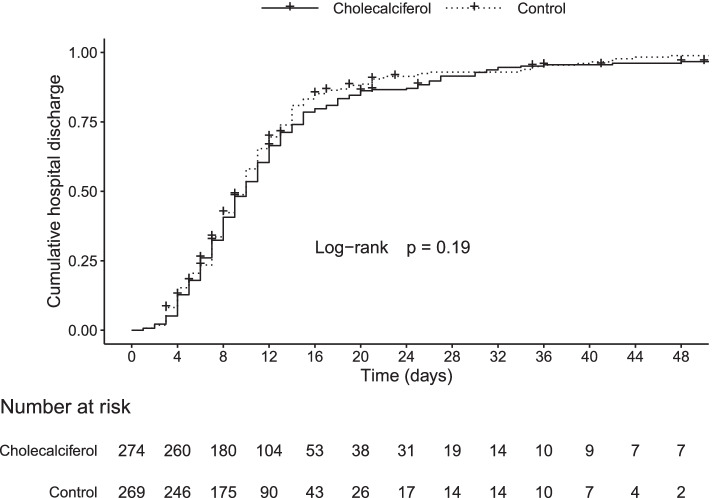
Cumulative hospital discharge in the cholecalciferol and control groups. Symbols represent censoring events

**Fig. 3:**
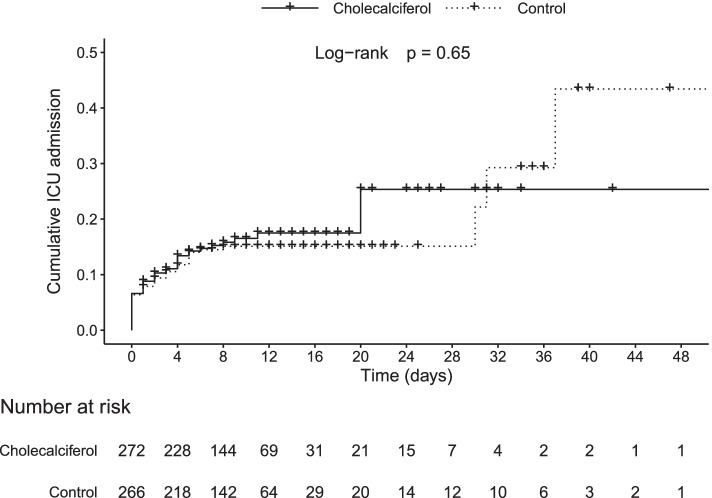
Cumulative ICU admission in the cholecalciferol and control groups. Symbols represent censoring events

**Fig. 4:**
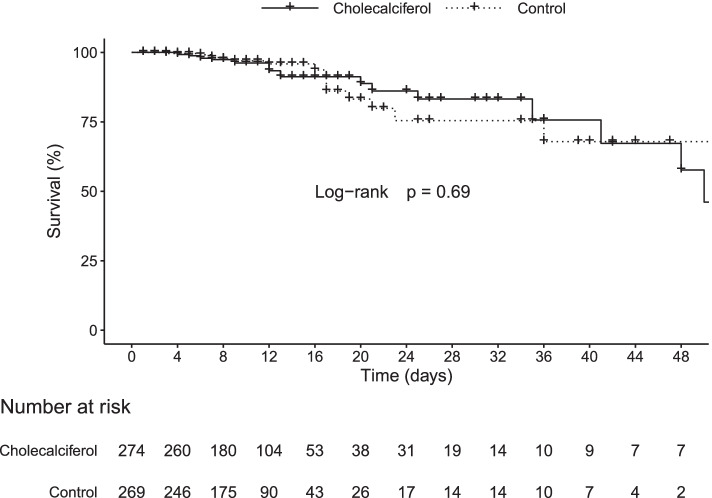
Kaplan-Meier estimates of survival in the cholecalciferol and control groups. Symbols represent censoring events

There were no differences in the three outcomes studied between the cholecalciferol and the control group; median length of hospitalisation 10.0 [95%CI 9.0–10.5] vs*.* 9.5 [95%CI 9.0–10.5] days, admission to ICU 17.2% [95%CI 13.0–22.3] vs*.* 16.4% [95%CI 12.3–21.4], and death 8.0% [95%CI 5.2–12.1] vs. 5.6% [95%CI 3.3–9.2], respectively (Figs. 2, 3 and 4). Thirty-seven patients died (22 in the cholecalciferol and 15 in the control groups). In the cholecalciferol group, the effect-modification by vitamin D levels was tested and there were no differences in outcomes related to the variation in serum calcidiol levels.

**Fig. 5:**
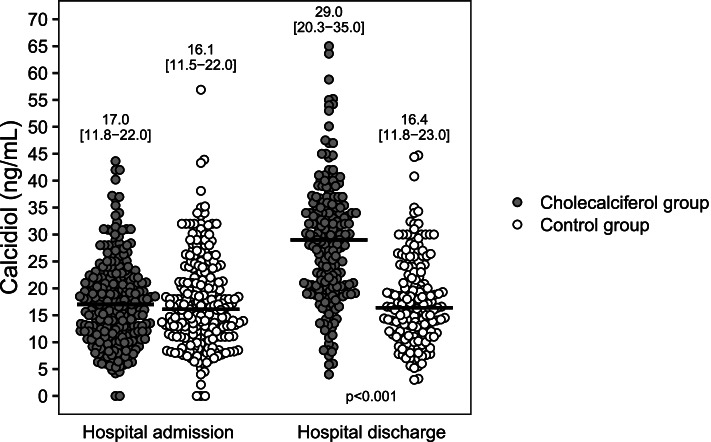
Calcidiol levels at hospital admission and discharge in the cholecalciferol and control groups. Horizontal lines show the median values per group. The numbers above the points show the median and [interquartile range]. Mann-Whitney *U* test was used to compare calcidiol levels at discharge

At hospital discharge, the most frequent symptoms were cough (28.9%), weakness (15.3%) and dyspnoea (13.6%) (Additional file 1: Table S2). In the cholecalciferol group, serum calcidiol was higher compared with the control group 29.0 vs. 16.4ng/mL, *p*=0.000), respectively. No other differences were observed in the biochemical parameters (Additional file 1: Table S3 and Fig. 5).

### Cohort analysis by calcidiol levels at hospital admission

Patients in the lowest calcidiol category (≤ 10 ng/mL) were older than patients in the higher category (> 25 ng/mL, Additional file 1: Table S4). In the five comorbidities analysed, no significant differences were observed among the calcidiol categories (Additional file 1: Table S4). Significant differences in C-reactive protein, serum albumin, haemoglobin, calcium and phosphate were found among the five calcidiol categories, but no differences were observed in the remaining parameters (Additional file 1: Table S5). Similar differences were found in the age-matched analyses (Additional file 1: Table S6).

A greater percentage of pulmonary involvement at admission was observed in the lowest compared with the highest calcidiol category (92.7% [95% CI 85.1–96.8] vs.70.1% [95%CI 59.2–79.2], Additional file 1: Table S7). A higher rate in the ICU admission was observed in patients with the lowest calcidiol levels, which was highly significant after age-matched analyses (Additional file 1: Table S7). There were no significant differences in the time of hospitalisation and death rate by calcidiol levels.

Serum calcidiol at admission >25 ng/mL was associated with a lower risk of pulmonary involvement at admission (OR 0.21[95%CI 0.08–0.60]), less days of hospitalisation (−3.69[95%CI −6.47–0.90] days) and lower risk of ICU admission (HR 0.35[95%CI 0.13–0.95]) compared with serum calcidiol ≤10 ng/mL after adjustment by demographics, comorbidities and laboratory parameters (Table 5). The associations remained significant after the age-matched analyses. There was no association between serum calcidiol and mortality (Table 5).

**Table 5:** Multivariate analysis of the association between serum calcidiol at admission and pulmonary involvement, length of hospitalization, admission to ICU and mortality

**Pulmonary involvement at hospital admission**^**a**^ **(binary logistic regression)**			
**Serum calcidiol >25 vs. ≤10 ng/mL**	***n***	**Odds ratio[95%CI]**	***p*-value**
Unadjusted	538	0.18[0.08–0.45]	<0.001
Adjusted by demographics	538	0.21[0.08–0.53]	0.001
Adjusted by demographics and comorbidities	538	0.20[0.08–0.51]	0.001
Adjusted by demographics, comorbidities and laboratory parameters	476	0.21[0.08–0.60]	0.003
Age-matched	325	0.25[0.08–0.74]	0.012
Patients not treated with cholecalciferol	235	0.14[0.03–0.59]	0.007
**Days of hospitalization (Linear regression)**			
**Serum calcidiol >25 vs. ≤10 ng/mL**	***n***	**Coefficient[95%CI]**	***p*-value**
Unadjusted	502	−4.08[−6.81–−1.36]	0.003
Adjusted by demographics	502	−3.69[−6.42–−0.96]	0.008
Adjusted by demographics and comorbidities	502	−3.64[−6.37–−0.90]	0.009
Adjusted by demographics, comorbidities and laboratory parameters	444	−3.69[−6.47–−0.90]	0.010
Age-matched	303	−4.41[−7.57–−1.25]	0.007
Patients not treated with cholecalciferol	222	−4.41[−8.09–−0.73]	0.020
**Admission to ICU (Cox regression)**			
**Serum calcidiol >25 vs. ≤10 ng/mL**	***n***	**Hazard ratio[95%CI]**	***p*-value**
Unadjusted	533	0.30[0.12−0.73]	0.008
Adjusted by demographics	533	0.33[0.13−0.82]	0.017
Adjusted by demographics and comorbidities	533	0.34[0.13−0.84]	0.019
Adjusted by demographics, comorbidities and laboratory parameters	471	0.35[0.13−0.95]	0.039
Age-matched	322	0.30[0.11−0.83]	0.021
Patients not treated with cholecalciferol	232	0.32[0.08−1.25]	0.101
**Mortality (Cox regression)**			
**Serum calcidiol >25 vs. ≤10 ng/mL**	***n***	**Hazard ratio[95%CI]**	***p*-value**
Unadjusted	538	1.10[0.39−3.08]	0.853
Adjusted by demographics	538	1.13[0.40−3.18]	0.810
Adjusted by demographics and comorbidities	538	1.32[0.44−3.91]	0.618
Adjusted by demographics, comorbidities and laboratory parameters	476	2.17[0.66−7.17]	0.205
Age-matched	325	1.90[0.51−7.11]	0.341
Patients not treated with cholecalciferol	235	4.99[0.74−33.45]	0.098

^a^Assessed by X-ray and/or computed axial tomography

Demographic variables: age and sex

Comorbidity variables: diabetes, cardiovascular disease, hypertension, asthma and chronic obstructive pulmonary disease

Laboratory parameters: C-reactive protein and leucocytes

Age-matched: age-matched patients by calcidiol categories. Adjusted by sex, comorbidities and laboratory parameters

Patients not treated with cholecalciferol: adjusted by demographics, comorbidities and laboratory parameters

Additional analyses can be found in the Additional file 1: Tables S8-S13.

## Discussion

The results of the trial showed that there were no differences in the outcomes of the COVID-19 disease between patients who received a single oral bolus of 100,000 IU of cholecalciferol at hospital admission compared with those who did not receive it. A cohort analysis showed that serum calcidiol at hospital admission was associated with outcomes.

As expected, demographics, comorbidities, pulmonary involvement, symptoms, biochemical parameters, serum calcidiol levels and types of drugs receive during the hospitalisation were well balanced in the cholecalciferol and control groups (Tables 2, 3 and 4). Even though a single dose of cholecalciferol achieved a significant increment of serum calcidiol level at discharge (+12.0 ng/ml), no differences in outcomes were observed.

Similar results to our study were obtained in a recently published Brazilian study in hospitalised patients with moderate-severe COVID-19 disease, in which the administration of 200,000 IU of cholecalciferol did not lead to reduction in hospital stay, mechanical ventilation, patients admitted to ICU and mortality [54]. However, this study had some limitations such as a higher prevalence of diabetes, hypertension and obesity in the group of patients that received vitamin D [55].

Both studies have similarities and differences, the more relevant were the duration of hospitalisation, 2.5 days shorter and the serum calcidiol at admission and discharge 4.3 ng/mL and 15.4 higher, respectively, compared with our study, likely due to the higher dose of cholecalciferol administered in the former (a single oral dose of 200,000 IU). In both trials, patients with COVID-19 disease who require hospitalisation, showed a significant increment in serum calcidiol during the hospital stay which was not able to render outcome benefits.

Apart from the two large trials discussed above, other open-label trial with lower number of participants (*n* = 76) has been published [15], but the authors did not provide information related with vitamin D status at baseline, in addition, the drug administration schedule and the formulation of vitamin D used was different to the Brazilian and our study. They used an activated form of vitamin D, (calcifediol −25(OH)D_3_−_,_ 0.532 mg administered orally on day one, followed by 0.266 mg on days three and seven, and then 0.266 weekly until discharge). The differences between both studies and the lower total number of participants, considering both studies together (*n* = 316) and deaths (*n* =17), prevented to combine them in further analyses and drew the attention to the importance of our study to investigate the role of vitamin D administered at hospital admission, in the management of COVID-19 disease.

The present COVID-VIT-D trial is so far the largest multicentre international trial designed to investigate the impact of the use of a single oral bolus of non-active vitamin D in clinical outcomes of moderate-severe COVID-19 disease in hospitalised patients, like the Brazilian trial [54], the result of the COVID-VIT-D trial was negative and similar results with the use of vitamin D have been observed in previous trials performed in other infectious diseases [18, 27, 56–60]. However, the lack of response of bolus versus daily dosing of vitamin D in several diseases, such as respiratory infections including the COVID-19 disease, is a matter of controversy [19, 61].

The results of the cohort analysis showed that higher calcidiol at admission was associated with less pulmonary involvement and better clinical outcomes. However, in the cohort analysis, there are multiple overlapping risk factors that can play an important role as confounders, such as age, diabetes, hypertension, cardiovascular disease, obesity and chronic obstructive pulmonary disease. Many of them were included in the multivariate adjustments, but still other non-measured confounders could have contributed to residual confounding. Furthermore, this cohort analysis may be subject to bias because the population recruited for the study was heterogeneous, i.e., different countries with uneven socioeconomic issues and health system coverage, and different latitudes that can influence calcidiol levels through different sun exposures [62].

According to the results of the cohort analyses, we could think that other factors such as the time that cholecalciferol may need to achieve its full modulatory function to reinforce the immune system could have played a positive role. In fact, a bolus dosing of 100,000 IU of cholecalciferol significantly increases serum calcidiol levels in a few days [23], but it may not be able to obtain the long-term systemic effects of calcitriol on the antimicrobial proteins such as cathelicidin, defensins or regulatory T cells [19, 23]. If this is the case, cholecalciferol should be given in advance, before the full COVID-19 disease is established, to promote a more effective immunological background for protection against the SARS-Cov-2 virus infection. However, this possible explanation remains in the speculative area.

The COVID-VIT-D study has some limitations; the time between the onset of symptoms and the administration of vitamin D was not analysed, and because it was an open label trial not controlled by placebo, it cannot be considered level-one evidence. However, the study has several important strengths, including its international nature (performed in 12 centres from four countries in two continents north and south of the equator), and the large number of patients recruited for the trial. The expertise of the HUCA Spanish coordinating centre in leading European and Latin American studies [63–65] was useful to design a study as simple and complete as possible, taking into account the difficulties of the pandemic and the strategic limitations of the participating centres.

## Conclusions

In summary, the results of the COVID-VIT-D trial demonstrated that in the moderate-severe COVID-19 disease that needs hospitalisation, a single oral bolus of cholecalciferol (100,000 IU), administered at admission did not improve the outcomes of the disease compared with patients who did not receive it. A cohort analysis showed that high serum calcidiol level at hospital admission was associated with better outcomes.

## Supplementary Information

**Additional file 1: Table S1.** Variables collected in the COVID-VIT-D trial. T. **Table S2.** Symptoms at discharge. **Table S3.** Biochemical parameters at discharge. **Table S4.** Demographic, comorbidities, and serum calcidiol categories at hospital admission. **Table S5.** Relevant biochemical parameters and serum calcidiol categories at hospital admission. **Table S6.** Relevant biochemical parameters and serum calcidiol categories at hospital admission in age-matched patients. **Table S7.** Pulmonary involvement at admission and outcomes according to serum calcidiol categories. **Table S8.** Types and number of drugs received during the hospitalization and serum calcidiol categories at hospital admission. **Table S9.** Demographic, comorbidities, and serum calcidiol categories at admission in age-matched patients. **Table S10.** Types and number of drugs received during the hospitalization and serum calcidiol categories at hospital admission in age-matched patients. **Table S11.** Relevant biochemical parameters and serum calcidiol categories at hospital admission in the control group (No cholecalciferol). **Table S12.** Types and number of drugs received during the hospitalization and serum calcidiol categories at hospital admission in the control group (No cholecalciferol). **Table S13.** Pulmonary involvement at admission and outcomes according to initial serum calcidiol categories in the control group (No cholecalciferol).

**Additional file 2.** Original trial protocol.

**Additional file 3.** COVID-VIT-D collaborators and affiliations.

## Data Availability

The data underlying this article will be shared upon reasonable request to the corresponding author.

## Abbreviations

AEMPs: Spanish Agency for Medicines and Health Products
BMI: Body mass index
CAT: Computed axial tomography
CRP: C-reactive protein
HUCA: Hospital Universitario Central de Asturias
ICU: Intensive care unit
IL-6: Interleukin 6
PCR: Polymerase chain reaction
SARS-CoV-2: Severe Acute Respiratory Syndrome CoronaVirus 2
