# Supplementary material for: A single-oral bolus of 100,000 IU of cholecalciferol at hospital admission did not improve outcomes in the COVID-19 disease: the COVID-VIT-D—a randomised multicentre international clinical trial
Source: BMC Med. 2022 Feb 18;20:83. doi: 10.1186/s12916-022-02290-8 (PMC8853840; doi:10.1186/s12916-022-02290-8)
Supplement: Supplementary file 1 — Additional file 1: Table S1. Variables collected in the COVID-VIT-D trial. T. Table S2. Symptoms at discharge. Table S3. Biochemical parameters at discharge. Table S4. Demographic, comorbidities, and serum calcidiol categories at hospital admission. Table S5. Relevant biochemical parameters and serum calcidiol categories at hospital admission. Table S6. Relevant biochemical parameters and serum calcidiol categories at hospital admission in age-matched patients. Table S7. Pulmonary involvement at admission and outcomes according to serum calcidiol categories. Table S8. Types and number of drugs received during the hospitalization and serum calcidiol categories at hospital admission. Table S9. Demographic, comorbidities, and serum calcidiol categories at admission in age-matched patients. Table S10. Types and number of drugs received during the hospitalization and serum calcidiol categories at hospital admission in age-matched patients. Table S11. Relevant biochemical parameters and serum calcidiol categories at hospital admission in the control group (No cholecalciferol). Table S12. Types and number of drugs received during the hospitalization and serum calcidiol categories at hospital admission in the control group (No cholecalciferol). Table S13. Pulmonary involvement at admission and outcomes according to initial serum calcidiol categories in the control group (No cholecalciferol). [file 12916_2022_2290_MOESM1_ESM.pdf]

ADDITIONAL FILE 1 COVID-VIT-D

A single oral dose bolus of 100,000 IU of cholecalciferol at hospital admission did not improve outcomes in the COVID-19 disease. The COVID-VIT-D: A randomized multicentre international clinical trial.

TABLE OF CONTENTS

- **Table S1.** Variables collected in the COVID-VIT-D trial.
- **Table S2.** Symptoms at discharge.
- **Table S3.** Biochemical parameters at discharge.
- **Table S4.** Demographic, comorbidities, and serum calcidiol categories at hospital admission.
- **Table S5.** Relevant biochemical parameters and serum calcidiol categories at hospital admission.
- **Table S6.** Relevant biochemical parameters and serum calcidiol categories at hospital admission in age-matched patients.
- **Table S7**. Pulmonary involvement at admission and outcomes according to serum calcidiol categories.
- **Table S8.** Types and number of drugs received during the hospitalization and serum calcidiol categories at hospital admission.
- **Table S9.** Demographic, comorbidities, and serum calcidiol categories at admission in age-matched patients.
- **Table S10.** Types and number of drugs received during the hospitalization and serum calcidiol categories at hospital admission in age-matched patients.
- **Table S11.** Relevant biochemical parameters and serum calcidiol categories at hospital admission in the control group (No cholecalciferol).
- **Table S12.** Types and number of drugs received during the hospitalization and serum calcidiol categories at hospital admission in the control group (No cholecalciferol).
- **Table S13.** Pulmonary involvement at admission and outcomes according to initial serum calcidiol categories in the control group (No cholecalciferol).

Table S1. Variables collected in the COVID-VIT-D trial.

Demographic and comorbidities	
Date of birth	Diabetes (yes/no)
Gender (male/female)	Cardiovascular disease (yes/no)
Height (cm)	Hypertension (yes/no)
Weight (kg)	Asthma (yes/no)
Smoking (yes/no)	COPD (yes/no)
ACEIs-ARBs use (yes/no)	
Hospitalization (clinical and evolutive data)	
Hospital admission date	Symptoms onset date
Hospital discharge date	Symptoms at admission
ICU admission date	Symptoms at discharge
ICU discharge date	Death date
Positive PCR SARS-COV2 date	
Biochemical and imaging parameters at admission and discharge	
Calcidiol (ng/mL)	Leucocytes (No./µL)
CRP (mg/dL)	Interleukin-6 (pg/mL)
Creatinine (mg/dL)	Ferritin (ng/mL)
Albumin (g/L)	Bilirubin (mg/dL)
Haemoglobin (g/dL)	Troponin (ng/L)
LDH (U/L)	Calcium (mg/dL)
D-dimer (ng/mL)	Phosphate (mgl/dL)
Procalcitonin (ng/mL)	X-Ray/CAT (positive/ doubtful/negative)
Symptoms at admission/discharge	
Cough (yes/no)	Anosmia (yes/no)
Fever (yes/no)	Diarrhoea (yes/no)
Weakness (yes/no)	Ageusia (yes/no)
Headache (yes/no)	Others (yes/no)
Dyspnoea (yes/no)	
Types of drugs received during the hospitalization	
Cholecalciferol (yes/no)	Enoxaparin (yes/no)
Azithromycin (yes/no)	Methylprednisolone (yes/no)
Ceftriaxone (yes/no)	Dexamethasone (yes/no)
Hydroxychloroquine (yes/no)	Interferon ß (yes/no)
Lopinavir/ritonavir (yes/no)	SARS-COV2 convalescent plasma (yes/no)
Tocilizumab (yes/no)	

ACEIs-ARBs: angiotensin converting enzyme inhibitors -angiotensin receptor blockers
COPD: Chronic obstructive pulmonary
CRP: C-reactive protein
CAT: Computed axial tomography

Table S2. Symptoms at discharge.

	Cholecalciferol group		Control group	
	**n**	**n=274**	**n**	**n=269**
**Symptoms**				
Fever, n (%)	234	8 (3.4)	237	18 (7.6)
Cough, n (%)	234	62 (26.5)	237	74 (31.2)
Weakness, n (%)	234	34 (14.5)	237	38 (16.0)
Dyspnoea, n (%)	234	33 (14.1)	237	31 (13.1)
Headache, n (%)	234	11 (4.7)	237	18 (7.6)
Anosmia, n (%)	234	11 (4.7)	237	9 (3.8)
Diarrhoea, n (%)	234	2 (0.9)	237	6 (2.5)
Ageusia, n (%)	234	2 (0.9)	237	5 (2.1)
Other, n (%)	234	4 (1.7)	237	8 (3.4)
Number of symptoms, median [IQR]	234	0.0 [0.0, 1.0]	237	1.0 [0.0, 1.0]

n: number of patients available for analysis
IQR: interquartile range

Table S3. Biochemical parameters at discharge.

	Cholecalciferol group		Control group	
	**n**	**n=274**	**n**	**n=269**
**Laboratory parameters**				
Calcidiol (ng/mL), median [IQR]	207	29.0 [20.3, 35.0]	187	16.4 [11.8, 23.0]
Creatinine (mg/dL), median [IQR]	116	0.9 [0.8, 1.0]	120	0.8 [0.7, 1.0]
CRP (mg/dL), median [IQR]	164	1.3 [0.4, 16.2]	176	1.2 [0.3, 9.6]
Albumin (g/L), median [IQR]	84	38.0 [35.0, 40.0]	90	39.0 [38.0, 41.0]
Haemoglobin (g/dL), median [IQR]	123	13.5 [12.6, 14.1]	122	13.9 [12.6, 14.6]
LDH (U/L), median [IQR]	89	310.0 [195.0, 424.0]	83	283.0 [196.0, 350.0]
Leucocytes (No./µL), median [IQR]	176	7.5 [6.3, 9.2]	176	7.3 [5.9, 8.8]
Interleukin-6 (pg/mL), median [IQR]	62	2.0 [0.9, 13.0]	52	2.0 [0.5, 6.5]
Ferritin (ng/mL), median [IQR]	110	649.4 [340.2, 993.5]	114	535.0 [277.2, 990.2]
Calcium (mg/dL), median [IQR]	113	9.0 [8.7, 9.3]	122	9.0 [8.7, 9.3]
Phosphate (mg/dL), median [IQR]	77	3.4 [3.0, 4.0]	90	3.5 [3.0, 3.9]

IQR: interquartile range
CRP: C-reactive protein
LDH: Lactate dehydrogenase

Table S4. Demographic, comorbidities, and serum calcidiol categories at hospital admission

Calcidiol levels (ng/mL)	All patients		≤10		10-15		15-20		20-25		>25		
	n	n=538	n	n=96	n	n=143	n	n=124	n	n=88	n	n=87	p-value
**Demographics**													
Age (years), median [IQR]	538	58.0 [46.0, 68.8]	96	62.0 [51.0, 75.2]*	143	57.0 [46.0, 67.0]	124	57.0 [46.0, 67.2]	88	60.0 [47.8, 72.0]**	87	56.0 [43.0, 65.0]	0.004
Males, n (%)	538	349 (64.9)	96	51 (53.1)	143	98 (68.5)	124	86 (69.4)	88	57 (64.8)	87	57 (65.5)	0.101
BMI (Kg/m²), median [IQR]	417	28.4 [25.7, 31.6]	62	28.7 [25.4, 32.9]	114	28.8 [26.4, 31.4]	102	28.1 [24.9, 32.3]	69	28.7 [26.8, 31.9]	70	27.1 [25.3, 29.8]	0.059
Smokers, n (%)	536	59 (11.0)	96	10 (10.4)	143	16 (11.2)	123	18 (14.6)	88	10 (11.4)	86	5 (5.8)	0.388
**Comorbidities**													
Hypertension, n (%)	538	234 (43.5)	96	49 (51.0)	143	59 (41.3)	124	57 (46.0)	88	40 (45.5)	87	29 (33.3)	0.158
Diabetes, n (%)	538	132 (24.5)	96	27 (28.1)	143	29 (20.3)	124	34 (27.4)	88	25 (28.4)	87	17 (19.5)	0.341
Cardiovascular disease, n (%)	538	112 (20.8)	96	27 (28.1)	143	29 (20.3)	124	27 (21.8)	88	17 (19.3)	87	12 (13.8)	0.205
Asthma, n (%)	538	28 (5.2)	96	10 (10.4)	143	5 (3.5)	124	7 (5.6)	88	2 (2.3)	87	4 (4.6)	0.136
COPD, n (%)	538	23 (4.3)	96	6 (6.2)	143	4 (2.8)	124	6 (4.8)	88	4 (4.5)	87	3 (3.4)	0.732

Kruskal-Wallis test was used to assess significant differences among the calcidiol categories for numeric variables
Chi-squared or Fisher exact test (frequencies less 5) were used for categorical variables
* p<0.005, calcidiol ≤10 vs. calcidiol 10-15, 15-20 and >25 ng/mL respectively (Mann-Whitney U test)
** p<0.03, calcidiol 20-25 vs. calcidiol >25 ng/mL (Mann-Whitney U test)
n: number of patients available for analysis
IQR: interquartile range
COPD: Chronic obstructive pulmonary disease

Table S5. Relevant biochemical parameters and serum calcidiol categories at hospital admission

Calcidiol levels (ng/mL)	All patients		≤10		10-15		15-20		20-25		>25		
	n	n=538	n	n=96	n	n=143	n	n=124	n	n=88	n	n=87	p-value
**Laboratory parameters**													
Calcidiol (ng/mL), median [IQR]	538	16.6 [11.6, 22.0]	96	8.0 [6.3, 8.6]	143	12.9 [11.5, 13.9]	124	18.0 [16.6, 18.8]	88	22.0 [21.0, 23.9]	87	30.0 [27.4, 32.0]	<0.001
Creatinine (mg/dL), median [IQR]	521	0.9 [0.8, 1.1]	91	0.9 [0.7, 1.2]	139	0.9 [0.7, 1.1]	121	0.9 [0.8, 1.1]	87	0.9 [0.8, 1.1]	83	0.9 [0.8, 1.1]	0.434
CRP (mg/dL), median [IQR]	477	9.4 [3.7, 30.0]	85	12.5 [3.8, 47.0]	128	11.7 [3.7, 35.2]	108	10.1 [4.0, 39.5]	79	7.7 [3.4, 18.4]	77	6.3 [3.4, 11.7]	0.012
Albumin (g/L), median [IQR]	297	39.0 [35.0, 41.0]	62	38.0 [35.0, 40.0]	83	40.0 [36.0, 41.0]	64	38.0 [36.0, 40.0]	49	38.0 [34.0, 40.0]	39	41.0 [38.5, 42.0]	0.003
Haemoglobin (g/dL), median [IQR]	527	13.9 [13.0, 14.8]	92	13.5 [12.5, 14.3]	139	13.8 [12.9, 14.4]	124	14.0 [12.9, 15.0]	87	14.0 [13.2, 14.8]	85	14.3 [13.2, 15.0]	0.005
LDH (U/L), median [IQR]	440	369.0 [248.8, 476.0]	76	356.0 [260.2, 449.0]	118	387.5 [280.0, 469.8]	101	388.0 [254.0, 497.0]	74	363.0 [234.8, 456.0]	71	299.0 [168.0, 518.0]	0.241
Leucocytes (No./µL), median [IQR]	527	7.0 [5.3, 9.1]	92	6.7 [4.8, 9.2]	139	7.3 [5.6, 9.6]	124	6.8 [5.3, 8.8]	87	7.4 [5.8, 9.4]	85	6.7 [5.0, 8.6]	0.279
Interleukin-6 (pg/mL), median [IQR]	188	12.1 [4.6, 27.0]	40	17.1 [7.0, 47.8]	42	13.0 [7.0, 21.0]	39	9.9 [4.2, 28.2]	32	11.1 [3.5, 25.8]	35	6.3 [3.6, 19.1]	0.096
Ferritin (ng/mL), median [IQR]	444	650.0 [340.8, 1286.8]	78	675.0 [389.0, 1259.0]	116	634.5 [343.8, 1127.0]	98	685.0 [382.5, 1257.1]	76	679.0 [337.8, 1359.8]	76	529.0 [257.0, 1553.0]	0.832
Calcium (mg/dL), median [IQR]	394	8.8 [8.5, 9.1]	59	8.7 [8.3, 8.9]	111	8.8 [8.4, 9.1]	89	8.8 [8.5, 9.1]	68	8.9 [8.5, 9.2]	67	8.9 [8.7, 9.2]	0.006
Phosphate (mg/dL), median [IQR]	310	3.3 [2.7, 3.9]	37	2.9 [2.6, 3.2]	76	3.0 [2.6, 3.6]	72	3.3 [2.8, 3.7]	63	3.5 [3.1, 4.0]	62	3.4 [3.0, 4.1]	<0.001

Kruskal-Wallis test was used to assess significant differences among the calcidiol categories
n: number of patients available for analysis
IQR: interquartile range
CRP: C-reactive protein
LDH: Lactate dehydrogenase

Table S6. Relevant biochemical parameters and serum calcidiol categories at hospital admission in age-matched patients.

Calcidiol levels (ng/mL)	All patients		≤10		10-15		15-20		20-25		>25		
	**n**	**n=365**	**n**	**n=73**	**n**	**n=73**	**n**	**n=73**	**n**	**n=73**	**n**	**n=73**	**p-value**
**Laboratory parameters**													
Calcidiol (ng/mL), median [IQR]	365	18.0 [11.4, 23.9]	73	8.1 [7.3, 8.9]	73	12.9 [11.4, 13.9]	73	18.0 [16.6, 18.9]	73	22.0 [21.0, 23.9]	73	29.6 [27.0, 32.0]	<0.001
Creatinine (mg/dL), median [IQR]	353	0.9 [0.8, 1.1]	71	0.9 [0.7, 1.1]	71	0.9 [0.7, 1.1]	70	0.9 [0.8, 1.1]	72	0.9 [0.8, 1.1]	69	0.9 [0.8, 1.2]	0.610
CRP (mg/dL), median [IQR]	325	8.1 [3.3, 24.0]	65	15.0 [4.9, 47.0]	65	8.7 [2.0, 27.0]	65	8.8 [3.3, 15.1]	66	6.7 [3.4, 17.0]	64	6.0 [3.5, 11.6]	0.019
Albumin (g/L), median [IQR]	203	39.0 [36.0, 40.8]	48	39.0 [36.0, 40.7]	43	40.0 [36.0, 41.0]	39	38.0 [36.0, 39.0]	41	38.0 [34.0, 40.0]	32	40.0 [37.2, 42.0]	0.029
Haemoglobin (g/dL), median [IQR]	358	13.9 [13.0, 14.7]	71	13.6 [12.7, 14.2]	71	13.8 [13.0, 14.4]	73	14.0 [12.9, 14.8]	72	14.0 [13.4, 14.8]	71	14.3 [13.2, 14.9]	0.058
LDH (U/L), median [IQR]	300	357.5 [246.8, 474.8]	63	371.0 [298.0, 473.5]	60	383.0 [276.5, 455.2]	59	312.0 [220.5, 510.5]	60	358.5 [226.0, 458.5]	58	318.0 [200.0, 528.0]	0.668
Leucocytes (No./µL), median [IQR]	358	6.9 [5.1, 9.0]	71	6.8 [4.8, 9.7]	71	7.5 [5.4, 9.6]	73	6.6 [5.0, 9.0]	72	7.3 [5.2, 9.0]	71	6.7 [5.0, 8.3]	0.568
Interleukin-6 (pg/mL), median [IQR]	141	12.1 [4.0, 27.0]	28	17.1 [7.0, 43.2]	27	13.0 [6.0, 20.0]	29	11.2 [4.0, 33.8]	28	12.6 [3.1, 26.2]	29	7.4 [3.9, 21.0]	0.369
Ferritin (ng/mL), median [IQR]	307	688.0 [342.2, 1318.0]	60	723.5 [423.2, 1494.8]	60	648.0 [378.6, 1109.0]	60	741.6 [374.5, 1144.5]	63	708.0 [344.7, 1340.5]	64	595.0 [272.8, 1645.5]	0.898
Calcium (mg/dL), median [IQR]	273	8.8 [8.5, 9.1]	49	8.7 [8.3, 8.9]	59	8.8 [8.4, 9.1]	53	8.8 [8.5, 9.0]	56	8.9 [8.5, 9.2]	56	8.9 [8.6, 9.2]	0.048
Phosphate (mg/dL), median [IQR]	224	3.3 [2.8, 3.8]	29	3.0 [2.6, 3.2]	44	3.2 [2.7, 3.6]	47	3.2 [2.7, 3.7]	52	3.5 [3.1, 3.9]	52	3.3 [2.8, 4.0]	0.017

Kruskal-Wallis test was used to assess significant differences among the calcidiol categories
n: number of patients available for analysis
IQR: interquartile range
CRP: C-reactive protein
LDH: Lactate dehydrogenase

Table S7. Pulmonary involvement at admission and outcomes according to serum calcidiol categories.

Calcidiol levels (ng/mL)	All patients		≤10		10-15		15-20		20-25		>25	
**ALL PATIENTS**	**n**	**n=538**	**n**	**n=96**	**n**	**n=143**	**n**	**n=124**	**n**	**n=88**	**n**	**n=87**
**Pulmonary involvement, %[95% CI]***	538	82.9[79.4-85.9]	96	92.7[85.1-96.8]	143	84.6[77.4-89.9]	124	80.6[72.4-87.0]	88	85.2[75.7-91.6]	87	70.1[59.2-79.2]
**Outcomes**												
Length of hospitalization (days), median [95% CI]	502	9.5[9.0-10.0]	86	11.5[10.0-13.5]	139	9.5[8.5-10.5]	112	9.5[8.5-10.5]	84	9.0[8.0-10.5]	81	9.5[8.5-10.5]
Admission to ICU, %[95% CI]	538	16.7[13.7-20.2]	96	24.0[16.1-34.0]	143	16.8[11.3-24.1]	124	18.5[12.4-26.7]	88	14.8[8.4-24.3]	87	8.0[3.6-16.4]
Death, %[95% CI]	538	6.7[4.8-9.2]	96	10.4[5.4-18.7]	143	2.8[0.9-7.5]	124	9.7[5.3-16.6]	88	4.5[1.5-11.9]	87	6.9[2.8-15.0]
**AGE-MATCHED PATIENTS**	**n**	**n=365**	**n**	**n=73**	**n**	**n=73**	**n**	**n=73**	**n**	**n=73**	**n**	**n=73**
**Pulmonary involvement, %[95% CI]***	365	85.8[81.6-89.1]	73	91.8[82.4-96.6]	73	87.7[77.4-93.9]	73	89.0[79.0-94.8]	73	89.0[79.0-94.8]	73	71.2[59.3-80.9]
**Outcomes**												
Length of hospitalization (days), median [95% CI]	340	9.5[9.0-10.0]	66	11.5[10.0-14.0]	71	9.5[8.0-11.0]	67	9.0[8.0-10.5]	69	9.0[7.5-11.0]	67	9.5[8.5-10.5]
Admission to ICU, %[95% CI]	365	17.3[13.6-21.6]	73	28.8[19.1-40.7]	73	15.1[8.1-25.8]	73	20.5[12.3-31.9]	73	13.7[7.1-24.2]	73	8.2[3.4-17.6]
Death, %[95% CI]	365	6.8[4.6-10.1]	73	9.6[4.3-19.3]	73	2.7[0.5-10.4]	73	8.2[3.4-17.6]	73	5.5[1.8-14.2]	73	8.2[3.4-17.6]

n: number of patients available for analysis
ICU: intensive care unit
*Assessed by chest X-ray and/or computed axial tomography

Table S8. Types and number of drugs received during the hospitalization and serum calcidiol categories at hospital admission.

Calcidiol levels (ng/mL)	All patients		≤10		10-15		15-20		20-25		>25		
	n	n=538	n	n=96	n	n=143	n	n=124	n	n=88	n	n=87	p-value
**Drugs prescribed**													
Cholecalciferol, n (%)	538	273 (50.7)	96	48 (50.0)	143	69 (48.3)	124	65 (52.4)	88	51 (58.0)	87	40 (46.0)	0.535
Enoxaparin, n (%)	529	396 (74.9)	92	74 (80.4)	141	110 (78.0)	122	88 (72.1)	87	68 (78.2)	87	56 (64.4)	0.080
Ceftriaxone, n (%)	530	191 (36.0)	92	39 (42.4)	141	47 (33.3)	123	49 (39.8)	87	30 (34.5)	87	26 (29.9)	0.361
Methylprednisolone, n (%)	531	191 (36.0)	92	37 (40.2)	141	42 (29.8)	123	44 (35.8)	88	28 (31.8)	87	40 (46.0)	0.112
Azithromycin, n (%)	532	183 (34.4)	92	39 (42.4)	141	39 (27.7)	124	39 (31.5)	88	29 (33.0)	87	37 (42.5)	0.073
Dexamethasone, n (%)	532	161 (30.3)	92	24 (26.1)	141	48 (34.0)	124	37 (29.8)	88	22 (25.0)	87	30 (34.5)	0.461
Number of drugs per patient, median [IQR]*	532	3.0 [2.0, 4.0]	92	3.0 [2.0, 4.0]	141	3.0 [2.0, 4.0]	124	3.0 [2.0, 4.0]	88	3.0 [2.0, 4.0]	87	3.0 [2.0, 4.0]	0.200

Kruskal-Wallis test was used to assess significant differences among the calcidiol categories for numeric variables
Chi-squared or Fisher exact test (frequencies less 5) were used for categorical variables
n: number of patients available for analysis
IQR: interquartile range
*Includes cholecalciferol

Table S9. Demographic, comorbidities, and serum calcidiol categories at admission in age-matched patients.

Calcidiol levels (ng/mL)	All patients		≤10		10-15		15-20		20-25		>25		
	n	n=365	n	n=73	n	n=73	n	n=73	n	n=73	n	n=73	p-value
**Demographics**													
Age (years), median [IQR]	365	58.0 [50.0, 67.0]	73	58.0 [50.0, 66.0]	73	57.0 [51.0, 67.0]	73	57.0 [51.0, 67.0]	73	59.0 [48.0, 69.0]	73	58.0 [51.0, 67.0]	0.990
Males, n (%)	365	227 (62.2)	73	35 (47.9)	73	49 (67.1)	73	50 (68.5)	73	47 (64.4)	73	46 (63.0)	0.076
BMI (Kg/m^2), median [IQR]	280	28.7 [26.0, 32.0]	50	28.9 [26.5, 32.9]	58	29.2 [26.8, 32.1]	59	28.7 [24.9, 32.9]	56	28.8 [26.8, 31.9]	57	27.7 [25.2, 29.9]	0.063
Smokers, n (%)	363	37 (10.2)	73	9 (12.3)	73	6 (8.2)	72	10 (13.9)	73	9 (12.3)	72	3 (4.2)	0.250
**Comorbidities**													
Hypertension, n (%)	365	157 (43.0)	73	35 (47.9)	73	32 (43.8)	73	35 (47.9)	73	29 (39.7)	73	26 (35.6)	0.490
Diabetes, n (%)	365	92 (25.2)	73	22 (30.1)	73	17 (23.3)	73	19 (26.0)	73	18 (24.7)	73	16 (21.9)	0.819
Cardiovascular disease, n (%)	365	70 (19.2)	73	14 (19.2)	73	17 (23.3)	73	17 (23.3)	73	10 (13.7)	73	12 (16.4)	0.500
Asthma, n (%)	365	15 (4.1)	73	8 (11.0)	73	1 (1.4)	73	2 (2.7)	73	2 (2.7)	73	2 (2.7)	0.067
COPD, n (%)	365	16 (4.4)	73	2 (2.7)	73	3 (4.1)	73	4 (5.5)	73	4 (5.5)	73	3 (4.1)	0.976

Kruskal-Wallis test was used to assess significant differences among the calcidiol categories for numeric variables
Chi-squared or Fisher exact test (frequencies less 5) were used for categorical variables
n: number of patients available for analysis
IQR: interquartile range
COPD: Chronic obstructive pulmonary disease

Table S10. Types and number of drugs received during the hospitalization and serum calcidiol categories at hospital admission in age-matched patients.

Calcidiol levels (ng/mL)	All patients		≤10		10-15		15-20		20-25		>25		
	**n**	**n=538**	**n**	**n=96**	**n**	**n=143**	**n**	**n=124**	**n**	**n=88**	**n**	**n=87**	**p-value**
**Drugs prescribed**													
Cholecalciferol, n (%)	538	273 (50.7)	96	48 (50.0)	143	69 (48.3)	124	65 (52.4)	88	51 (58.0)	87	40 (46.0)	0.535
Enoxaparin, n (%)	529	396 (74.9)	92	74 (80.4)	141	110 (78.0)	122	88 (72.1)	87	68 (78.2)	87	56 (64.4)	0.080
Ceftriaxone, n (%)	530	191 (36.0)	92	39 (42.4)	141	47 (33.3)	123	49 (39.8)	87	30 (34.5)	87	26 (29.9)	0.361
Methylprednisolone, n (%)	531	191 (36.0)	92	37 (40.2)	141	42 (29.8)	123	44 (35.8)	88	28 (31.8)	87	40 (46.0)	0.112
Azithromycin, n (%)	532	183 (34.4)	92	39 (42.4)	141	39 (27.7)	124	39 (31.5)	88	29 (33.0)	87	37 (42.5)	0.073
Dexamethasone, n (%)	532	161 (30.3)	92	24 (26.1)	141	48 (34.0)	124	37 (29.8)	88	22 (25.0)	87	30 (34.5)	0.461
Number of drugs per patient, median [IQR]*	532	3.0 [2.0, 4.0]	92	3.0 [2.0, 4.0]	141	3.0 [2.0, 4.0]	124	3.0 [2.0, 4.0]	88	3.0 [2.0, 4.0]	87	3.0 [2.0, 4.0]	0.200

Kruskal-Wallis test was used to assess significant differences among the calcidiol categories for numeric variables
Chi-squared or Fisher exact test (frequencies less 5) were used for categorical variables
n: number of patients available for analysis
IQR: interquartile range
*Includes cholecalciferol

Table S11. Relevant biochemical parameters and serum calcidiol categories at hospital admission in the control group (No cholecalciferol).

Calcidiol levels (ng/mL)	All patients		≤10		10-15		15-20		20-25		>25		
	n	n=265	n	n=48	n	n=74	n	n=59	n	n=37	n	n=47	p-value
**Laboratory parameters**													
Calcidiol (ng/mL), median [IQR]	265	16.1 [11.5, 22.0]	48	8.0 [7.0, 8.5]	74	13.0 [11.5, 13.9]	59	17.8 [16.6, 18.4]	37	22.0 [21.1, 24.0]	47	30.0 [27.6, 32.0]	<0.001
Creatinine (mg/dL), median [IQR]	253	0.9 [0.8, 1.1]	44	0.9 [0.7, 1.1]	71	0.9 [0.7, 1.1]	57	0.9 [0.8, 1.1]	37	0.9 [0.8, 1.1]	44	0.9 [0.8, 1.2]	0.861
CRP (mg/dL), median [IQR]	236	8.7 [3.3, 25.0]	39	15.0 [3.8, 55.5]	66	14.2 [2.3, 42.8]	53	9.7 [3.0, 23.0]	35	6.6 [2.7, 11.9]	43	6.3 [4.5, 9.9]	0.017
Albumin (g/L), median [IQR]	143	39.0 [36.6, 41.0]	26	39.0 [36.2, 40.0]	42	40.0 [38.2, 41.8]	29	38.0 [36.0, 39.0]	24	38.0 [35.8, 39.2]	22	41.0 [40.0, 42.0]	0.003
Haemoglobin (g/dL), median [IQR]	258	14.0 [13.0, 14.9]	45	13.5 [12.7, 14.3]	71	14.0 [13.0, 14.6]	59	14.2 [12.7, 15.0]	37	14.0 [13.4, 14.9]	46	14.4 [13.6, 15.1]	0.027
LDH (U/L), median [IQR]	212	347.0 [243.0, 461.0]	35	390.0 [298.0, 522.0]	61	384.0 [255.0, 476.0]	47	390.0 [280.5, 464.5]	30	274.5 [194.8, 376.2]	39	291.0 [93.5, 421.5]	0.006
Leucocytes (No./µL), median [IQR]	258	7.0 [5.1, 8.9]	45	7.5 [5.0, 10.5]	71	7.5 [5.6, 9.4]	59	6.8 [5.2, 8.2]	37	6.8 [5.1, 8.9]	46	6.7 [4.9, 8.7]	0.726
Interleukin-6 (pg/mL), median [IQR]	91	11.0 [3.8, 24.9]	16	19.5 [7.0, 51.0]	17	13.0 [6.1, 21.1]	18	8.9 [3.8, 25.2]	18	14.7 [3.4, 25.3]	22	5.7 [3.2, 12.1]	0.205
Ferritin (ng/mL), median [IQR]	216	584.0 [302.8, 1108.5]	39	650.0 [305.5, 1151.0]	56	465.0 [292.2, 872.5]	45	793.0 [453.0, 1300.0]	33	514.0 [231.0, 1166.0]	43	452.0 [297.0, 917.5]	0.156
Calcium (mg/dL), median [IQR]	190	8.9 [8.5, 9.1]	29	8.6 [8.3, 8.9]	58	8.9 [8.6, 9.1]	44	8.8 [8.5, 9.1]	25	9.1 [8.9, 9.3]	34	8.9 [8.7, 9.2]	0.004
Phosphate (mg/dL), median [IQR]	149	3.2 [2.7, 3.8]	21	2.9 [2.6, 3.1]	36	3.0 [2.6, 3.5]	33	3.3 [3.1, 3.6]	24	3.6 [3.1, 4.0]	35	3.7 [3.0, 4.1]	0.004

Kruskal-Wallis test was used to assess significant differences among the calcidiol categories
n: number of patients available for analysis
IQR: interquartile range
CRP: C-reactive protein
LDH: Lactate dehydrogenase

Table S12. Types and number of drugs received during the hospitalization and serum calcidiol categories at hospital admission in the control group (No cholecalciferol).

Calcidiol levels (ng/mL)	All patients		≤10		10-15		15-20		20-25		>25		
	n	n=265	n	n=48	n	n=74	n	n=59	n	n=37	n	n=47	p-value
**Drugs prescribed**													
Cholecalciferol, n (%)	265	0 (0.0)	48	0 (0.0)	74	0 (0.0)	59	0 (0.0)	37	0 (0.0)	47	0 (0.0)	–
Enoxaparin, n (%)	260	187 (71.9)	45	33 (73.3)	73	54 (74.0)	58	45 (77.6)	37	28 (75.7)	47	27 (57.4)	0.181
Ceftriaxone, n (%)	260	92 (35.4)	45	18 (40.0)	73	24 (32.9)	58	19 (32.8)	37	15 (40.5)	47	16 (34.0)	0.868
Methylprednisolone, n (%)	261	92 (35.2)	45	17 (37.8)	73	22 (30.1)	59	21 (35.6)	37	11 (29.7)	47	21 (44.7)	0.510
Azithromycin, n (%)	261	95 (36.4)	45	19 (42.2)	73	20 (27.4)	59	16 (27.1)	37	16 (43.2)	47	24 (51.1)	0.032
Dexamethasone, n (%)	261	78 (29.9)	45	14 (31.1)	73	20 (27.4)	59	20 (33.9)	37	9 (24.3)	47	15 (31.9)	0.855
Number of drugs per patient, median [IQR]*	261	3.0 [3.0, 4.0]	45	4.0 [3.0, 5.0]	73	3.0 [2.0, 4.0]	59	3.0 [3.0, 4.0]	37	4.0 [3.0, 4.0]	47	3.0 [3.0, 4.0]	0.379

Kruskal-Wallis test was used to assess significant differences among the calcidiol categories for numeric variables
Chi-squared or Fisher exact test (frequencies less 5) were used for categorical variables
n: number of patients available for analysis
IQR: interquartile range
*Includes cholecalciferol

Table S13. Pulmonary involvement at admission and outcomes according to initial serum calcidiol categories in the control group (No cholecalciferol).

Calcidiol levels (ng/mL)	All patients		≤10		10-15		15-20		20-25		>25	
	**n**	**n=265**	**n**	**n=48**	**n**	**n=74**	**n**	**n=59**	**n**	**n=37**	**n**	**n=47**
**Pulmonary involvement, %[95% CI]***	265	80.4[75.0-84.9]	48	91.7[79.1-97.3]	74	82.4[71.5-90.0]	59	79.7[66.8-88.6]	37	83.8[67.3-93.2]	47	63.8[48.5-76.9]
**Outcomes**												
Length of hospitalization (days), median [95% CI]	251	9.5[9.0-10.0]	42	11.5[9.5-14.5]	74	9.0[8.0-10.5]	54	9.0[8.0-10.5]	37	8.5[7.0-10.0]	44	10.0[8.5-11.0]
Admission to ICU, %[95% CI]	265	16.2[12.1-21.3]	48	22.9[12.5-37.7]	74	18.9[11.1-30.0]	59	22.0[12.7-35.1]	37	5.4[0.9-19.5]	47	6.4[1.7-18.6]
Death, %[95% CI]	265	5.3[3.0-8.9]	48	12.5[5.2-25.9]	74	0.0[0.0-6.1]	59	8.5[3.2-19.4]	37	0.0[0.0-11.7]	47	6.4[1.7-18.6]

n: number of patients available for analysis
ICU: intensive care unit
*Assessed by chest X-ray and/or computed axial tomography
