# Supplementary material for: A single-oral bolus of 100,000 IU of cholecalciferol at hospital admission did not improve outcomes in the COVID-19 disease: the COVID-VIT-D—a randomised multicentre international clinical trial
Source: BMC Med. 2022 Feb 18;20:83. doi: 10.1186/s12916-022-02290-8 (PMC8853840; doi:10.1186/s12916-022-02290-8)
Supplement: Supplementary file 2 — Additional file 2. Original trial protocol. [file 12916_2022_2290_MOESM2_ESM.pdf]

Supplement 1. Trial protocol and statistical analysis plan

Title: Usefulness of vitamin D on morbidity and mortality of SARS-CoV-2 virus infection (Covid-19) at the Central University Hospital of Asturias

Principal Investigator:

Jorge Cannata Andía

Unidad de Gestión Clínica de Metabolismo Óseo

Hospital Universitario Central de Asturias (HUCA).

Instituto de Investigación Sanitaria del Principado de Asturias (ISPA)

Universidad de Oviedo

The Spanish version of this protocol was provisionally approved by the Research Ethics Committee of the Principality of Asturias on March 27, 2020 and definitively approved on April 28, 2020. It was authorized as a low-interventional clinical trial by the Spanish Agency of Medicines and Medical Devices (AEMPS) (EudraCT 2020-002274-28); on May 19, 2020.

INDEX

1. BACKGROUND 3

2. HYPOTHESIS AND OBJECTIVES 4

3. EFFICACY 4

4. METHODOLOGY, PATIENTS, INCLUSION AND EXCLUSION CRITERIA 4

5. DATA COLLECTION, LOGISTICS AND CHRONOLOGY 5

6. ANALYSIS OF RESULTS 6

7. ADVERSE EFFECTS 6

8. SAFETY ANALYSIS 7

9. ETHICAL ASPECTS 7

10.PROMOTER 8

11.ACCESS TO DATA 9

12.SCOPE 9

13.SCHEME OF THE TRIAL 9

14.ANNEXE 10

1. BACKGROUND

The effect of vitamin D on bone metabolism is the oldest, the best known and the most studied aspect of the action of this hormonal system (1,29,30). However, in the last two decades other important actions of vitamin D, called “non-classical” or pleiotropic effects, have been described (29,30). Vitamin D can exert its effect on multiple organs and tissues, since its receptor (VDR) is expressed in more than 30 tissues, it is considered that VDR activation regulates about 3% of the genome (29).

The “non-classical” actions of vitamin D are exerted at different levels, including cancer and the cardiovascular system. In the first, it has been used in the treatment of psoriasis and as an adjuvant in different neoplasms due to its antiproliferative action. An association between insufficient levels of vitamin D and poor evolution of the disease has been described in several tumours such as breast, colorectal, and prostate (31,32). At the cardiovascular level, benefits on vascular calcification and heart failure have been suggested (17,33), although other studies have not been conclusive (13,18,34-36).

Other areas of special interest related to this project are those that refer to the effects of vitamin D on inflammatory, immunological and infectious processes (3,5,6,29,37-40). Vitamin D is involved in the modulation of the immune system and reduces the expression of inflammatory mediators being used as adjuvant therapy in different types of infections such as tuberculosis and HIV (1, 38-40). The action of vitamin D is through many mechanisms (1, 39-41). Regarding the objective of this project, very recent studies indicate that the protein SKp2, -which vitamin D inhibits-, exacerbates autoimmune diseases and facilitates the replication of the coronavirus (42,43).

Vitamin D insufficiency is very common in general population (8,44). A study funded by the European Union carried out in general population randomly selected within the city of Oviedo showed that the prevalence of vitamin D insufficiency (calcidiol <18ng / mL) was 72% in the population aged 55-65 years and 80% in those over 65 years (8,30); results that were consistent with other studies from Spain and Europe (44,45). Vitamin D insufficiency is more prevalent in patients with cancer, tuberculosis, HIV infection, malnutrition, especially in those over 75 years of age, and in highly vulnerable patients with severe diseases (10,46,47). In these groups, it might be especially indicated to administer vitamin D (11) since several studies that have shown association between insufficient levels of vitamin D and mortality (12,48-53).

The clinical information of the “non-classical” effects of vitamin D is based almost exclusively on the information provided by observational studies; thus, the aim of this randomized clinical trial is to assess the effect of vitamin D supplementation on the clinical evolution and survival of hospitalized patients due to SARS-CoV-2 infection.

2. HYPOTHESIS AND OBJECTIVES

A large percentage of patients infected by SARS-CoV-2 may have insufficient vitamin D levels and consequently a poorer response to SARS-CoV-2 infection, especially those patients over 70 years of age in whom the disease has shown to be more lethal. The administration of vitamin D (cholecalciferol) could improve the clinical evolution of the disease and possibly decrease the mortality due to Covid-19 disease.

Objective:

The objective of this study is to assess whether the administration of a single oral dose of native vitamin D (100,000 IU of Cholecalciferol) (1 drinkable ampoule), can influence the evolution of clinical and biochemical parameters of the Covid-19 disease.

3. EFFICACY

The efficacy of the use of vitamin D, -in addition to the standard treatment-, will be compared with the standard treatment measuring the parameters detailed below:

Patients admitted to hospital

- Percentage of patients that become negative for SARS-CoV-2.
- Clinical symptoms during hospitalization.
- Improvement of biochemical and molecular parameters of inflammation
- Days of hospitalization
- Percentage of patients requiring admission to the intensive care unit (ICU)
- Average stay in the ICU
- Mortality during follow-up

4. METHODOLOGY, PATIENTS, INCLUSION AND EXCLUSION CRITERIA

Clinical trial in patients with SARS-CoV-2 infection who meet the inclusion criteria

a) Inclusion criteria

- Patients admitted to the HUCA Covid-19 Hospitalization Units
- Diagnosis of Covid-19 demonstrated by PCR positive for SARS-CoV-2 prior to randomization.
- Age> = 18 years
- Agreement to participate in the study (informed consent)

b) Exclusion criteria

- If discharge or fatal outcome is expected within 48 hours.
- Obvious cognitive impairment (inability to communicate)
- Negative PCR test for SARS-CoV-2 despite radiological, analytical and clinical findings compatible with the Covid-19 disease.

- Allergy to vitamin D
- Patients who are receiving or have received any form of vitamin D in the last 3 months
- Pregnant women

After acceptance, patients will be randomized to receive one of the two treatments.

*** Group 1:** 100,000 IU of Colecalciferol in a single oral dose (plastic ampoule)

*** Group 2:** No vitamin D

5. DATA COLLECTION, LOGISTICS AND CHRONOLOGY

Positive patients for the SARS-CoV-2 will be included in the study. They will be asked for their participation in the study and informed consent will be obtained verbally, stating their acceptance in the patient's medical records.

Patients admitted to hospital. If the randomization and assignment to one of the two treatment groups was not carried out in the emergency room, it will be carried out in the ward using the computer generated randomization list provided for that purpose (see data base table in the attached document).

In all cases, a database designed specifically for the study will be used identified by a code to preserve confidentiality, in accordance with the Spanish Law 3/2018 of December 5,2018 on the protection of personal data.

In this database, patient's baseline and follow-up data will be recorded. The databases of the outpatient facility and hospitalization ward include demographics and comorbidities, clinical symptoms, radiological and biochemical studies, treatments, admission to the ICU, including the date of each one (see database in attached document).

After randomization, 2 samples of blood will be drawn: **a)** 8mL gel clot activator tube or 100x16 mm without anticoagulant for the serum quantification of calcidiol (25 Hydroxyvitamin D) and general lab parameters (Laboratory, HUCA) and **b)** K3-EDTA collection tube, two tubes of 5ml 100x13 m/L or 1 tube of 10ml 100x13 m/L, for quantification of inflammation markers. The blood tubes will be coded with the same number assigned to the patient in the database. The plasma from tube b will be stored and kept in the Biobank of the Principality of Asturias.

Patients in both treatment arms will receive the same standard therapy for Covid-19 established by the Hospital policy.

** A second sample of blood will be collected before hospital discharge for the measurement of calcidiol.*

** A "Monitoring Committee" will be responsible of the evolution of the trial.*

6. ANALYSIS OF RESULTS

A. An interim analysis of results will be carried out separately for each group of patients (outpatient and hospitalization) when 60 patients (30 per group) have completed at least **14 days (interim control 1) or 21 days (interim control 2)** after randomization. In both cases, the response to treatment between groups 1 and 2 will be compared according to the efficacy criteria described in section 4.

B. Depending on the interim analysis, the aspects of the study that are considered necessary could be modified

C. The study will end when the last patient hospitalized for Covid-19 is discharged from hospital

D. The Monitoring Committee will assess the need and/or usefulness of prolonging the follow-up of 1 to 3 months after discharge in order to assess evolution and possible relapses.

7. ADVERSE EFFECTS

The number and percentage of patients dropping out due to adverse events and serious adverse events will be recorded. According the Spanish and European law that regulates clinical trial (RD 1090/2015), adverse effects are defined as follows:

Adverse event (AE); Any untoward medical occurrence in a patient or clinical investigation subject administered a pharmaceutical product and which does not necessarily have to have a causal relationship with the treatment. It will be the responsibility of the investigator to provide relevant information on the AE, its duration, relationship with the clinical trial, the beginning and end of the episode and its final result.

Serious adverse event (SAE); It is any incident harmful to health that, at any dose, makes hospitalization necessary or its prolongation, produces permanent or significant disability or disability, gives rise to a congenital anomaly or malformation, endangers life or causes death. It will be the responsibility of the researcher to report any SAE both to the Ethical Research Committee, to the relevant Authorities, as well as to provide the necessary information relevant to the SAE and its evolution. Following current regulations, the follow-up of patients who withdraw from the study due to an AAG should be until their complete recovery or stabilization.

Unexpected serious adverse reactions (USAR); It is a serious adverse reaction whose nature, severity or outcome are not consistent with the reference safety information and therefore not consistent with what is expected based on the known characteristics and technical data sheet of the product used in the study. It will be the responsibility of the researcher to inform the Ethical Research Committee and the relevant Authorities within 15 days from the knowledge of

the USAR, issuing a written report on it and EudraVigilance will be notified in accordance with RD 1090/2015.

All suspicions of AEs, SAEs, USARs, and important safety incidents that occur in the course of the clinical trial will be recorded in the "Notification of suspected adverse reactions" sheet that is attached in Annex 2 and the AEMPS and the Ethical Research Committee will be notified according to the procedures previously described in this document.

8. SAFETY ANALYSIS

The safety variables, cumulative incidence (95% CI) of AEs, SAEs and USARs of patients who have experienced at least one adverse event of any type throughout the follow-up period will be presented in table format.

9. ETHICAL ASPECTS

The trial will be carried out in accordance with the requirements of the Declaration of Helsinki (Hong Kong revision, September 1989) and following the recommendations of the European Union Good Clinical Practice (document 111/3976/88, July 1990) and current Spanish regulations.

This study has a patient information sheet and an informed consent document. However, in studies carried out with Covid-19 patients, it is a better option to request verbal consent in order to avoid the use of paper and contaminated material. For this reason, a written consent has been designed that is read to the patient prior to inclusion in the study. It will be recorded in the Electronic Health Record that all the information regarding the trial has been explained to the patient or her representatives, or legal guardian, and that the patient agrees to participate in it.

<u>The verbal information given to patients is as follows:</u>

You are invited to participate in this clinical trial on the effect of vitamin D on SARS-CoV2 infection. Vitamin D has been associated with benefits at the infectious level, there are no randomized clinical studies that have demonstrated its efficacy. For this reason, you are being invited to participate in a study aimed to find out the usefulness of vitamin D in controlling the infection of SARS-CoV-22 (Covid-19 disease) in order to reduce its adverse effects. For the registration of the study, a database created specifically for the study will be used. Data will be collected anonymously to guaranty confidentiality in accordance with Spanish Law on the Protection of Personal Data (3/2018 of December). This database will record who received vitamin D and who did not and if the patient was sent to home or was hospitalized.

Group 1: 100,000 IU of Cholecalciferol (Vitamin D) in a single oral dose (drinkable ampoule)

Group 2: No vitamin D

At the time of randomization, 2 tubes of 5 ml of blood will be drawn, one for the serum quantification of calcidiol (25 Hydroxyvitamin D) and general biochemistry that will be measured in the Biochemistry laboratory of the Central University Hospital of Asturias and in the other, markers of inflammation to be measured in the Bone Metabolism Laboratory of the Central University Hospital of Asturias. These blood samples will be kept and used anonymously only for research purpose.

Before giving your permission, you can ask all the questions you need to understand the details about the study and you can decide not to participate in this study. This decision will not affect the relationship with the health care team attending you or the healthcare to which you are entitled. The Principal Investigator of the study is Jorge Cannata–Andía from the Bone and Mineral Research Department at the Hospital Universitario Central de Asturias, Spain.

- Right to revoke the Informed Consent:
    - If you change your mind, you may request to be excluded from the study at any time. You can exercise your rights of access, rectification, cancellation Clinical Trials Department of the Hospital Universitario Central de Asturias, Spain. Avenida de Roma, s / n - 33011- Oviedo. Telephone: 34 985 108 083
- This request for participation in the clinical trial, as well as your response, will be recorded in your clinical records.
- Thank you for the attention you have given us,
- Person who reports
    - name / surname….
    - Title ...
    - Date …

10. PROMOTER

The Foundation for Biosanitary Research and Innovation of the Principality of Asturias (FINBA) is a non-profit organization that pursues general interest purposes and for this it is endowed with its own legal personality and full legal capacity to act. FINBA is developed as the management structure of the Principality of Asturias Health Research Institute (ISPA) grouped around the Hospital Universitario Central de Asturias, Spain. (HUCA), and also as a R&D&I entity for all health centres of the Principality of Asturias Health Service (SESPA).

11. ACCESS TO DATA

In order to guarantee the confidentiality of the trial data, only the researcher, his team of collaborators, the trial monitor and the Clinical Research Ethics Committee of the corresponding centre or the one supervising the trial and the authorities will have access to them. All data obtained in the study will be stored with a code and in a safe place, with restricted access. Throughout the process, confidentiality will be guaranteed in accordance with Spanish Organic Law 3/2018, of December 5, on the Protection of Personal Data and the guarantee of digital rights as well as the Regulation (EU) 2016/679 of the European Parliament and of the Council of April 27, 2016 on Data Protection.

12. SCOPE

The trial will be coordinated between HUCA and the Principality of Asturias Health Research Institute (ISPA-FINBA). In the near future this project could be implemented in other hospitals that consider it feasible and of interest.

The **Monitoring Committee** will be PI of the trial, the Coordinators of the outpatient facility and hospitalization ward, the head of the Pharmacy Department, the head of the Virology Section and a representative of the Clinical Research Ethics Committee. This Committee will be responsible for all practical aspects related to the execution of the trial.

13. SCHEME OF THE TRIAL

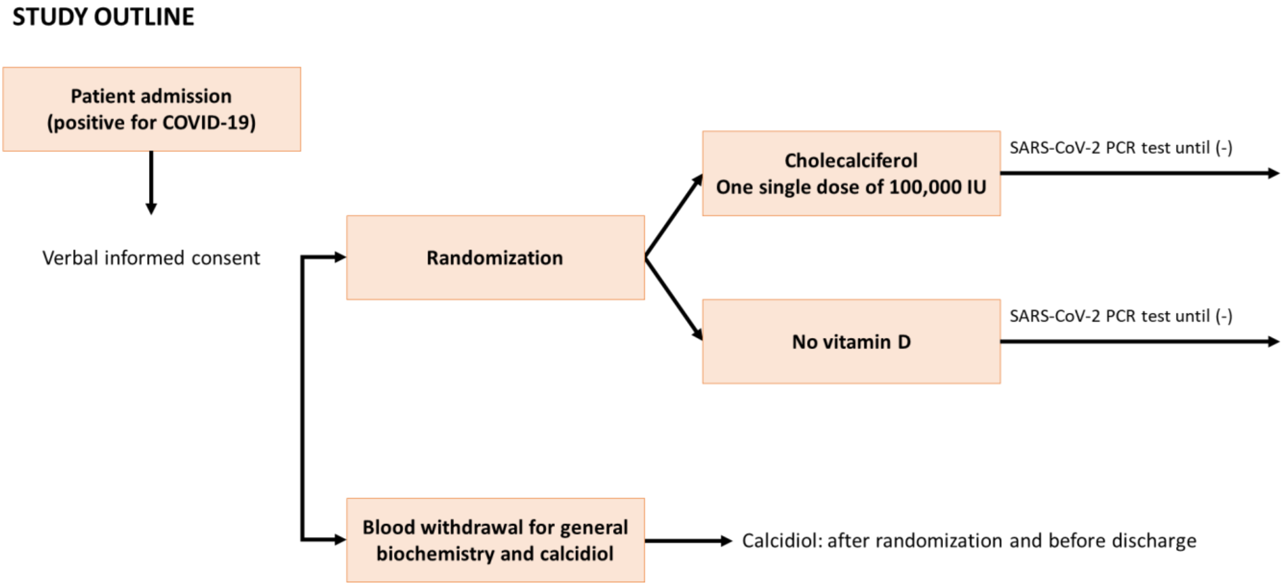

14. ANNEXE

NOTIFICATION OF SUSPECTED ADVERSE EVENTS

NOTIFICATION OF SUSPECTED ADVERSE EVENTS	PROTOCOL NUMBER:	NOTIFICATION NUMBER:
	PATIENT NUMBER:	
**I. INFORMATION ABOUT THE ADVERSE EVENT**		
1. INITIALS:	2. AGE:	3. SEX:
CONSEQUENCES:		
4. DESCRIPTION OF THE ADVERSE EVENT		
**II. PRODUCT INFORMATION UNDER INVESTIGATION**		
5. NAME:		
6. DAILY DOSE:		
7. ROUTE OF ADMINISTRATION:		
8. DISEASE UNDER STUDY:		
9. TREATMENT DATES:		
**III. CLINICAL HISTORY AND CONCOMMITANT MEDICINES**		
10. CONCOMITANT MEDICINES AND DATE OF ADMINISTRATION:		
11. IMPORTANT INFORMATION OF THE CLINICAL HISTORY (diagnostics, allergies, pregnancy, etc.)		
**IV. INFORMATION ABOUT PROMOTER AND INVESTIGATOR**		
12. NAME AND ADDRESS OF THE PROMOTER:		
13. NAME AND ADDRESS OF INVESTIGATOR:		
14. REPORT DATE:		
15. TYPE OF REPORT:	INITIAL	
	FOLLOW-UP	
