# Supplementary material for: A single-oral bolus of 100,000 IU of cholecalciferol at hospital admission did not improve outcomes in the COVID-19 disease: the COVID-VIT-D—a randomised multicentre international clinical trial
Source: BMC Med. 2022 Feb 18;20:83. doi: 10.1186/s12916-022-02290-8 (PMC8853840; doi:10.1186/s12916-022-02290-8)
Supplement: Supplementary file 3 — Additional file 3. COVID-VIT-D collaborators and affiliations. [file 12916_2022_2290_MOESM3_ESM.pdf]

ADDITIONAL FILE 3 COVID-VIT-D

A single oral dose bolus of 100,000 IU of cholecalciferol at hospital admission did not improve outcomes in the COVID-19 disease. The COVID-VIT-D: A randomized multicentre international clinical trial.

TABLE OF CONTENTS

- COVID-VIT-D collaborators and affiliations.

COVID-VIT-D collaborators and affiliations.

COUNTRY	NUMBER OF PATIENTS	CENTRE	COLLABORATOR
SPAIN	176	Hospital Universitario Central de Asturias (HUCA). Oviedo.	Alonso Montes, Cristina[1,2,3]<br>Álvarez Ramos, Belén[1,2]<br>Avello Llano, Noelia[1]<br>Díaz López, Bernardino[1]<br>Fernández Villabrille, Sara[1,2,3,4]<br>García Gil-Albert, Carmen[1]<br>García Lamigueiro, Carolina[1]<br>González Franco, Álvaro[1]<br>Gutiérrez Alcalá, Octavio[1]<br>Gutiérrez Rodríguez, José[2,5]<br>Lobo Cortizo, Isabel[1]<br>Martín Carro, Beatriz[1,2,3,4]<br>Martín Vírgala, Julia [1,2,3,4]<br>Martínez Arias, Laura[1,2,4]<br>Prieto García, María Belén[1]<br>Prieto Piquero, Eugenia[1]<br>Rodríguez Carrio, Javier[2,3,4]<br>Vázquez Fernández, Carlos[1]
		Hospital Universitario San Agustín. Avilés.	de Zárraga Fernández, Miguel Alberto[6]<br>Tarrazo Tarrazo, Carlos[6]
		Hospital universitario El Bierzo. Ponferrada.	de Alba García, Paula[7]<br>Fernández Crespo, Silvia[7]<br>Fernández García, Carolina[7]
		Hospital Lucus Augusti. Lugo.	Ayuso García, Blanca[8]
ARGENTINA	297	Hospital Privado Universitario de Córdoba y Hospital Raúl Ángel Ferreyra. Córdoba.	Alaye, María Luján[9,10]<br>Albertini, Ricardo[9,10,11]<br>Caeiro, Juan Pablo[9]<br>Capra, Raúl Horacio[9]<br>Garay, María Gabriela[9]<br>Saad, Emanuel José[9,10,11]
		Hospital Militar Central Cirujano Mayor Dr. Cosme Algerich. Buenos Aires.	López Martínez, Martín Nicolás[12]<br>Sánchez, Yamila Elizabeth[12]<br>Stessens, Maria Aline[12]
		Hospital Independencia. Santiago del Estero.	Agüero, Javier[13]<br>Cejas, Raúl[13]<br>Dorado, Andrea[13]<br>Secco, Pablo[13]<br>Vechetti, Guillermo[13]<br>Vergara, Ramón[13]
		Hospital Julio C. Perrando. Resistencia.	Díaz, Rafael[14]<br>Ramírez Farías, Rocío[14]
		Instituto de Nefrología Pergamino SRL. Pergamino.	Díaz, Romina[15]<br>Guerrero, María Paula[15]
GUATEMALA	47	Hospital General de Enfermedades del Instituto Guatemalteco de Seguridad Social (IGSS). Ciudad de Guatemala.	Ávalos, Lester[16]<br>Cipriano Maldonado, Ever[16]<br>Loaiza Espinales, José[16]
CHILE	28	Hospital Barros Luco Trudeau. Santiago.	Gacitúa Meneses, Ignacio[17,18]<br>Villalobos Navarro, Arturo[17,18]

Collaborators affiliations:

[1] Hospital Universitario Central de Asturias (HUCA), Oviedo, España.
[2] Instituto de Investigación Sanitaria del Principado de Asturias (ISPA), Oviedo, España.
[3] Retic REDinREN-ISCIII
[4] Universidad de Oviedo, Oviedo, España.
[5] Hospital Monte Naranco, Oviedo, España.
[6] Hospital Universitario San Agustín (HUSA), Avilés, España.
[7] Hospital Universitario El Bierzo, Ponferrada, España.
[8] Hospital Lucus Augusti, Lugo, España.
[9] Hospital Privado Universitario de Córdoba, Córdoba, Argentina.
[10] Hospital Raúl Ángel Ferreyra, Córdoba, Argentina.
[11] Instituto Universitario de Ciencias Biomédicas de Córdoba (IUCBC), Córdoba, Argentina.
[12] Hospital Militar Central Cirujano Mayor Dr. Cosme Argerich, Buenos Aires, Argentina.
[13] Hospital Independencia, Santiago del Estero, Argentina.
[14] Hospital Julio C. Perrando, Resistencia, Argentina.
[15] Instituto de Nefrología Pergamino SRL, Pergamino, Argentina.
[16] Hospital General de Enfermedades del Instituto Guatemalteco de Seguridad Social (IGSS), Ciudad de Guatemala, Guatemala.
[17] Hospital Barros Luco Trudeau, Santiago, Chile.
[18] Universidad de Chile, Santiago, Chile.
